# DNA methylation similarities in genes of black South Africans with systemic lupus erythematosus and systemic sclerosis

**DOI:** 10.1186/s12929-015-0142-2

**Published:** 2015-05-20

**Authors:** Puleng Matatiele, Mohamed Tikly, Gareth Tarr, Mary Gulumian

**Affiliations:** Toxicology & Biochemistry Research Section, National Institute for Occupational Health, National Health Laboratory Service, Johannesburg, South Africa; Division of Rheumatology, Faculty of Health Sciences, University of the Witwatersrand, Johannesburg, South Africa; Division of Molecular Medicine and Haematology, School of Pathology, Faculty of Health Sciences, University of the Witwatersrand, Johannesburg, South Africa

**Keywords:** Methylation, Peripheral blood, Genomic DNA, Systemic lupus erythematosus, Systemic sclerosis, Autoimmunity, Methyl qPCR arrays

## Abstract

**Background:**

Systemic lupus erythematosus (SLE) and systemic sclerosis (SSc) are systemic autoimmune connective tissue diseases that share overlapping clinico-pathological features. It is highly probable that there is an overlap in epigenetic landscapes of both diseases. This study aimed to identify similarities in DNA methylation changes in genes involved in SLE and SSc. Global DNA methylation and twelve genes selected on the basis of their involvement in inflammation, autoimmunity and/or fibrosis were analyzed using PCR arrays in three groups, each of 30 Black South Africans with SLE and SSc, plus 40 healthy control subjects.

**Results:**

Global methylation in both diseases was significantly lower (<25 %) than in healthy subjects (>30 %, p = 0.0000001). In comparison to healthy controls, a similar gene-specific methylation pattern was observed in both SLE and SSc. Three genes, namely; *PRF1*, *ITGAL and FOXP3* were consistently hypermethylated while CDKN2A and CD70 were hypomethylated in both diseases. The other genes (*SOCS1, CTGF, THY1, CXCR4, MT1-G, FLI1,* and *DNMT1*) were generally hypomethylated in SLE whereas they were neither hyper- nor hypo-methylated in SSc.

**Conclusions:**

SSc and SLE patients have a higher global hypomethylation than healthy subjects with specific genes being hypomethylated and others hypermethylated. The majority of genes studied were hypomethylated in SLE compared to SSc. In addition to the commonly known hypomethylated genes in SLE and SSc, there are other hypomethylated genes (such as *MT-1G and THY-1*) that have not previously been investigated in SLE and SSc though are known to be hypermethylated in cancer.

## Background

Systemic lupus erythematosus (SLE, lupus) and systemic sclerosis (SSc, scleroderma) are both autoimmune connective tissue diseases associated with autoantibody production. In SLE, inflammation often affecting more than one organ is the outstanding pathological feature [1], whereas in SSc immune activation results mainly in fibrosis of the skin and internal organs, and damage of small blood vessels [2,3]. Although the exact genetic causes of both SSc and SLE are still unknown, the environmental influence reflected by the epigenetic mechanisms, with DNA methylation changes in particular, are generally considered as key players in the onset and progression of both diseases [1,4,5]. The concordance of these conditions in monozygotic twins indicates that epigenetic factors, mediated by environmental factors, may have a role to play [4,6,7].

**Fig. 1:**
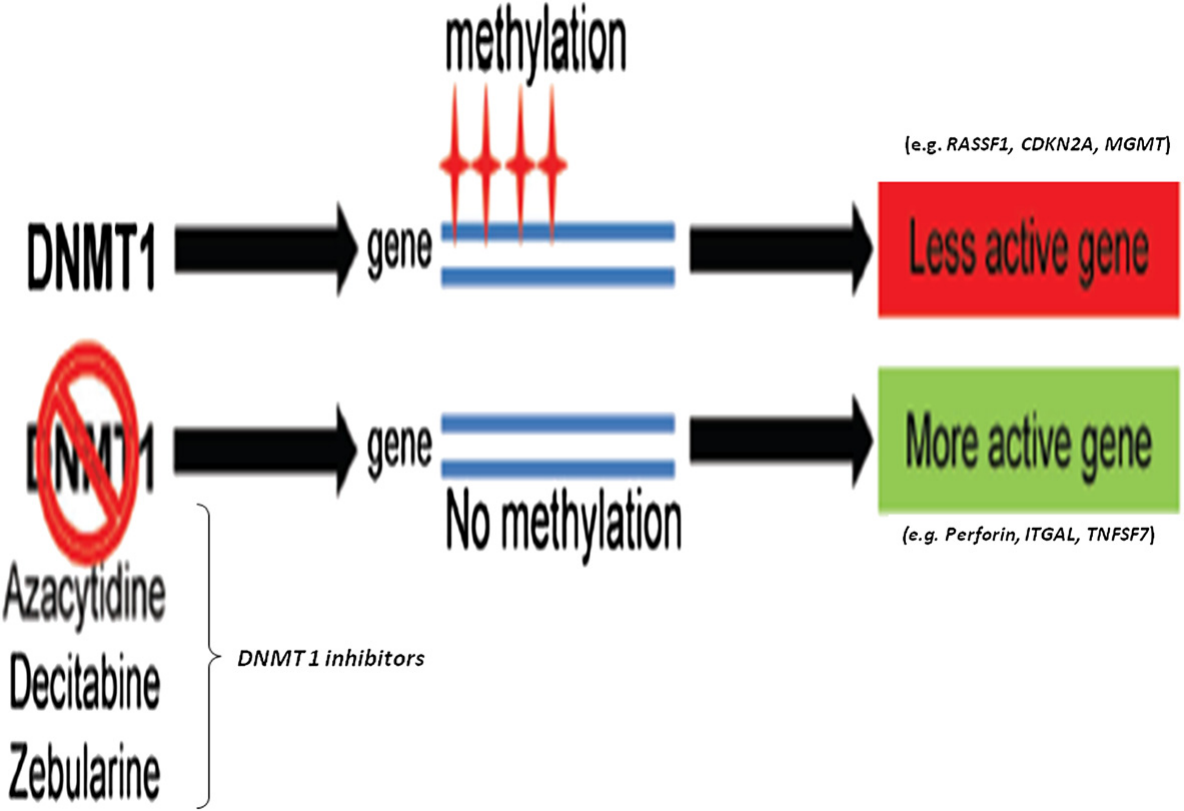
*Short title*: Role of *DNMT1* in DNA methylation. *Long title*: Methylation of gene promoters by *DNMT1* leads to gene silencing, while its inhibition leads to hypomethylation. Modified from Zielske, [66]

Epigenetic mechanisms, one example of which is DNA methylation, are vital for the development and function of the immune system. DNA methylation is important in the regulation of inflammatory genes [8]. Hypermethylation of promoter regions of genes is typically associated with transcriptional silencing while hypomethylation facilitates gene expression. In autoimmunity, aberrant DNA methylation profiles in genes encoding metalloproteinases, proinflammatory cytokines and chemokines, all processes that regulate inflammation have been reported. Although the roles and interactions of abnormal DNA methylation in relation to inflammation and immunity are not yet clear, evidence suggests that key mediators of inflammation-induced DNA methylation changes are oxidative stress and the increased pro-inflammatory cytokines [9]. Endogenous triggers such as antigens released from dying cells are recognized as the main stimulus to abnormal production of type I interferon (IFN-I) resulting in chronic inflammation in SLE and SSc, and perhaps other autoimmune diseases [10]. The clearance of apoptotic cells is impaired in SLE and SSc, providing a potential continuous source of endogenous antigen. Proper control of DNA methylation is maintained by the DNA methyltransferases (DNMTs), and it appears that persistent exposure to pro-inflammatory cytokines can contribute to DNA hypomethylation through decreased expression of DNA methyltransferase (*DNMT*) [9,11]. On the other hand, overexpression of the *de novo* methyltransferases is implicated in the establishment of gene-specific hypermethylation [12-14]. An illustration as to how methylation of gene promoters by *DNMT1* leads to gene silencing, while its inhibition leads to hypomethylation is shown in Fig. 1.

Accumulating evidence indicates that abnormal DNA hypomethylation and expression of methylation-related genes in CD4+ T cells are an important epigenetic hallmark associated with SLE and SSc [15-17]. This is accounted for by the fact that *DNMT1* expression and enzymatic activity is reduced in SLE and SSc, and given the high prevalence of inflammation and oxidative stress in both conditions [9,15]. Several classic methylation-sensitive autoimmunity-related genes in SLE and SSc have been identified that include *CD11a* (*ITGAL*), *perforin* (*PRF1*), *CD70* (*TNFSF7*), *CD40* ligand (*TNFSF5*) and *PP2Acα* [5,18]. Other top-ranked methylation-sensitive autoimmunity-related genes known to be associated with SSc include *CTGF*, *FLI1 and DNMT* [19,20]*.* In fact, current epigenetic studies reveal a growing list of genes dysregulated by altered DNA methylation in many autoimmune diseases. The array of genes dysregulated by altered DNA methylation thus provides an opportunity to examine the patterns of inactivation of such genes among different autoimmune diseases.

The aim of this study was to determine global DNA methylation and gene-specific methylation of potentially “overlapping” genes involved in either collagen synthesis, the inflammatory response or tumour suppression, in black African SLE and SSc patients using genomic DNA from whole blood. The choice of whole blood over CD4+ T cells as study material in this work was based on the consideration that SLE and SSc display many abnormalities across all arms of the immune system, represented in whole blood. In fact, SLE and SSc therapies attempting to target specific components of the immune system have so far not been successful, so much that broad-based immunosuppression still remains the mainstay in the treatment of both conditions [21]. Also, it has been confirmed that defects in epigenetic regulation of both CD4+ T cells [22,23] and B-cells [24-26] are involved in both SLE and SSc. Moreover, literature indicates that the total number of B and T lymphocytes is significantly reduced in SLE and SSc [27,28], a situation that is aggravated by the immunosuppression therapy. It has therefore become clear that the global methylation landscape in these two diseases involves both T and B lymphocytes, hence the choice to study global DNA methylation as opposed to methylation of only CD4+ T cells as in many other studies. Also, the great majority of patients in this study had been receiving glucocorticoids and immunosuppressants at different doses, and therefore it would have been unethical and not practically viable to collect enough blood from them to be able to study individual lymphocyte populations, hence the use of whole blood.

## Results

The clinical information for the study participants is presented in Tables 1 and 2. The records of all SLE and SSc patients enrolled in the study were retrospectively reviewed. In the case of SLE, patients’ disease-related symptoms reflecting disease severity such as skin and musculoskeletal involvement, serositis, systemic vasculitis and kidney involvement were identified. The data showed that these SLE patients fell into three phenotypic subsets as follows; 30 % (7 of 30) made SLE1 group (skin and musculoskeletal involvement); 57 % (17/30) formed SLE2 (serositis, systemic vasculitis, with no kidney involvement) and 13 % (4/30) made up SLE3 group (glomerulonephritis). All SSc patients had positive anti-nuclear autoantibodies (ANA) but only a few were positive for anti-centromere autoantibodies (ACA) and/or anti-topoisomerase antibodies (ATA). The SLEDAI score showed that SLE was either mild or moderate, with no severe cases of the condition in the study group. In addition, correlation of PGA score (not shown), SLEDAI and methylation status, showed that the sicker the patient (increasing SLEDAI and PGA score), the greater was the percentage of global hypomethylation as well as more number of genes demethylated. The majority (67 %, 20/30) of SLE patients also had discoid lupus erythematosus (DLE), while 50 % of them tested positive for the Coomb’s test.

**Table 1:** Demographics for study participants

Study subjects	Controls		SLE		SSc	
	n	Age range (mean) yrs	n	Age range (mean) yrs	n	Age range (mean) yrs
Male	12	20-51 (35)	5	22-66 (45)	3	48-56 (51)
Female	28	22-53 (36)	25	22-63 (42)	27	32-60 (47)
Total	40	20-53 (38)	30	22-66 (42)	30	32-60 (48)

**Table 2:** Lupus and scleroderma disease presentation according to autoantibody serology, manifestations and tests

Systemic Lupus Erythematosus (SLE)					Systemic Sclerosis (SSc)				
(n = 30)					(n = 30)				
Autoantibodies, manifestations and tests	SLEDAI score				Autoantibodies and manifestations	dcSSC(n = 14)	lcSSc(n = 5)	“other” (e.g. UCTD, morphea, Reynaud’s) (n = 3)	Unclassified(n = 7)
	*Mild, score <10(n = 21)*	*Moderate, score 10–20 (n = 3)*	*Severe, score >20 (n =0)*	*unscored (n = 6)*	ANA positive (n = 22) 73 %	13	6	5	n/a
ANA positive (n = 29) 97 %	20	3	0	6	ACA positive (n = 6) 20 %	3	2	1	n/a
DLE positive (n = 19) 63 %	15	2	0	3	ATA positive (n = 2) 7 %	2	0	0	n/a
Coombs’ test positive (n = 14) 47 %	9	3	0	3	ILD (n = 10) 33 %	6	1	3	n/a

*Abbreviations: ACA, anticentromere autoantibody; ANA, anti-nuclear antibodies; ATA, Antitopoisomerase antibodies; Coombs’ test, detects presence of antibodies that act against the surface of red blood cells, indicates haemolytic anaemia; DLE, Discoid lupus erythematosus; ILD, interstitial lung disease, indicator of direct pulmonary involvement, and leading cause of death; dcSSc, diffuse cutaneous systemic sclerosis; lcSSc, limited cutaneous systemic sclerosis; UCTD, undifferentiated connective* tissue disease; *SLEDAI, Systemic Lupus Erythematosus Disease Activity Index; n/a, results not available*.

**Fig. 2:**
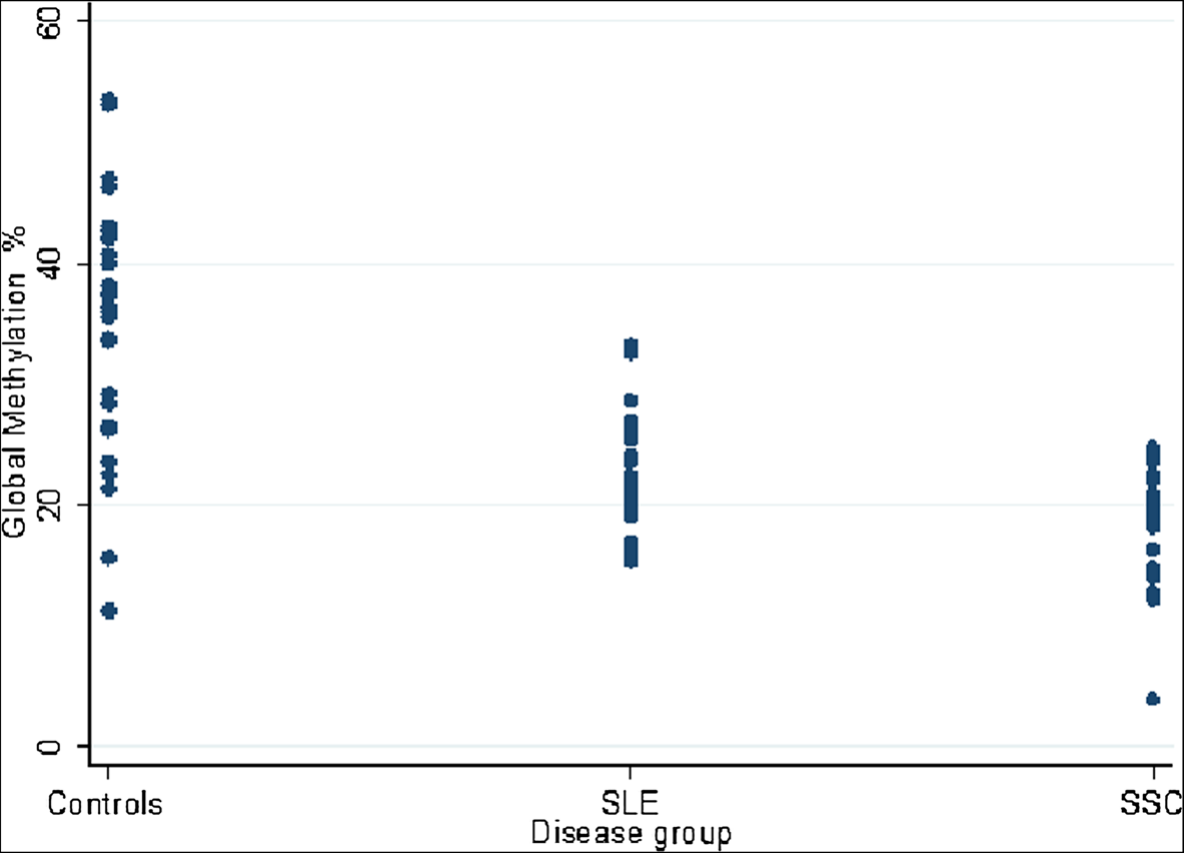
*Short title*: Global methylation in peripheral blood of SLE, SSc and healthy (C) subjects. A significant difference in global methylation between the study groups is indicated by the p-values. *Long Title*: Analysis of global methylation levels in peripheral blood of SLE, SSc and healthy control subjects (triplicate measurements). The dot plot shows a significant difference in global methylation between the patients and healthy subjects, which is also indicated by the p-values. However, SLE and SSc patients were not significantly different from each other although there was a trend towards lower values in the SSc group compared to the SLE group

**Fig. 3:**
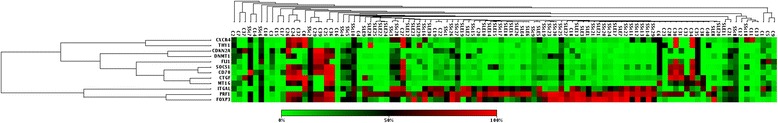
*Short title*: Comparison of gene-specific methylation in lupus (SLE), scleroderma (SSc) and healthy subjects (C). *Long title*: Heat map showing distribution of methylation among the 12 genes analyzed in lupus (SLE) and scleroderma (SSc) patients in comparison to healthy controls (C)

The SSc and SLE groups exhibited significantly lower global methylation levels, compared to the control group (p < 0.000001) (Fig. 2). This is so despite the fact that global hypomethylation was also observed even among some of the healthy subjects. Further analysis of the individual genes, as shown in the heat map (Fig. 3), indicates that the majority of genes included in the present study were hypomethylated in the patient groups [9 of 12 (75 %) of genes] compared to the control group. Conversely, three genes were hypermethylated in the patient groups, namely; *PRF1*, *ITGA*L and *FOXP3*. When comparing the two patient groups to each other a significantly differential methylation pattern was observed amongst the individual genes with the exception of *ITGAL* and *PRF1* which were consistently hypermethylated in both diseases while *CDKN2A* and *CD70* were consistently hypomethylated. Fig. 3 also shows a uniform methylation distribution among SLE patients, whereas SSc patients seem to split into several groups, which could perhaps indicate the different disease grades. All the other genes analyzed (*SOCS1, CTGF, THY1, CXCR4, MT1-G, FLI1,* and *DNMT1*) were generally hypomethylated in SLE whereas they were neither hypermethylated nor hypomethylated in most SSc patients.

## Discussion

Our findings confirm that DNA methylation is globally reduced in SLE and SSc, and that there is abnormal expression of methylation-related genes [15]. Also, increasing SLEDAI and PGA scores were indicative of increasing number of genes demethylated in SLE and worsening of the disease in the patient. In SSc there was no difference observed in both global and gene-specific DNA methylation between diffuse and limited sclerosis patients, even though subset-specific DNA methylation abnormalities have been reported between the two disease subtypes [29]. In fact both were equally hypomethylated (about 18 % DNA methylation). This highlights the robustness of genome-wide analyses over analysis of a few candidate genes in this study. Global hypomethylation was also observed even among some of the healthy subjects (Fig. 2). An important factor to consider in this situation is the well known fact that there are differences in methylation status of individuals which is to some extent controlled by certain host and lifestyle characteristics such as age, smoking, alcohol drinking, diet and health history [30-32]. These factors were controlled to a very limited extend in this study. Fig. 3 also shows that there are some patients that fall within both SLE and SSc groups. Serology tests (ANA positivity) also confirm overlap in both conditions (Table 3). This is not surprising since it is known that both SSc and SLE show an overlap of symptoms and in many instances there are patients who develop both conditions [17,33].

**Table 3:** Analysis of variance was conducted on the global methylation results with disease group as the factor variable, and the results were verified with Bartlett’s test. A Bonferroni correction shows that the SLE and SSc groups were significantly different from the controls but not significantly different from each other, although there was a trend towards lower values in the SSc group compared to the SLE group

Study groups	Global methylation %		
	*Mean*	*Standard deviation (SD)*	*Range*
Controls	33.9	11	11-53
SLE	22.2	5	15 – 33
SSc	17.7	5	3.8 - 24
Study groups comparison			
	*(mean difference) P value*		
	Controls	SLE	
SLE	(−11) p = 0.00	-	
SSc	(− 16) p = 0.00	(−4) p = 0.068	

In this study specific genes that were found to be hypomethylated include *CTGF*, *CXCR4*, *CD70, MT-1G*, *THY1, CDKN2A, SOCS1, FLI1* and *DNMT1. CTGF* is known to be constitutively upregulated in SSc and has been hypothesized to be a key mediator of pulmonary fibrosis due to its overexpression which correlates well with severity of lung fibrosis frequently observed in this disease [12]. Therefore, the observed CTGF hypomethylation in this study could well indicate the progression of both SLE and SSc to a stage of pulmonary involvement. In fact, the indication of pulmonary involvement, which is the presence of interstitial lung disease (ILD), was observed in 17 % (5/30) of the SSc patients. *CXCR4* has been found prominently, particularly in various leukocyte subsets of the skin and kidney of SLE patients, and has been shown to mediate chemotaxis of T-lymphocytes [34]. Hypomethylation of this gene in the study could also indicate its overexpression and hence progression of disease in these patients.

Hypermethylation results in suppression of *PRF1*, *ITGA*L and *FOXP3* and this explains why these three genes may have a role in the immunopathogenesis of SSc and SLE. For example, it has been demonstrated that the epigenetic control of *FOXP3* for effective function of T-regulatory cells dictates the requirement of a hypomethylated *FOXP3* promoter [35]. However, the opposite is seen in the case of these SLE and SSc patients, and this situation is known to correlate highly with parameters of disease severity and the high incidence of SLE and SSc and other autoimmune diseases.

Many studies report hypomethylation in promoter regions of *PRF1* and *ITGAL* with consequent overexpression of these genes in SLE. It is therefore tempting to attribute the observed hypermethylation of these two genes in this study to technical/experimental errors. However, further survey of literature indicates that there are high levels of free polyamines in SLE and that polyamines not only cause SLE, but they are also important in sustaining the disease [36,37]. These increased polyamine concentrations result in enhanced methylation of the *ITGAL* promoter and increased *DNMT1* activity [38]. Hypermethylation of the *ITGAL* promoter region has also been observed in CD4+ T cells from other autoimmune diseases [39]. So, we suggest that even though hypomethylated *ITGAL* and *PRF1* promoters are mostly reported as characteristic of SLE, this is not always the case as was seen in this study, and this situation may represent the co-occurrence of other autoimmune conditions such as diabetes and cancer, with SLE and SSc. The opposite from what is reported in other studies is also observed for several of these genes in this study. For example, hypermethylation of both *FLI1* in SSc and *SOCS1* in SLE was not observed in our study even though hypermethylation of promoter regions of both genes has been previously implicated in the pathophysiology of SSc and SLE, respectively [40-42]. However, *FLI-1* overexpression, probably as a result of promoter hypomethylation, has been detected in various types of cancer and other diseases [43]. Most importantly, it has been reported that in lupus-prone mice *Fli1* expression fails to become down-regulated likely due to aberrant transcriptional regulation [44]. Also, it is reported that increasing *SOCS1* expression by cells may be useful as a strategy to block CD8(+) T cell-mediated autoimmunity and to more generally prevent cytokine-dependent tissue destruction in inflammatory diseases [45]. The observed hypomethylation of *FLI1* and *SOCS1* could therefore imply that both genes were overexpressed in these patients for the reasons already mentioned. Similarly, other studies have reported that *CDKN2A* and *DNMT1* gene promoters are hypermethylated in DNA derived from plasma and blood cells of patients with SLE, whereas the opposite was observed in this study [46,47]. Increased expression of *CDKN2A* has been shown to be as a result of promoter demethylation [48,49] and recently it has been shown that presence of up-regulated *CDKN2A* expression, promotes apoptosis and cellular senescence of bone marrow-derived mesenchymal stem cells observed in SLE patients [50]. In the same way, Liu *et al*., [51] reported that expression of *DNMT1* mRNA was significantly increased in SLE, whereas other studies [52] describe the opposite. As mentioned above high levels of natural polyamines in SLE result in increased *DNMT1* activity, and this is probably due to a hypomethylated promoter region as was observed in this study.

The other genes (*MT-1G* and *THY1*) which were found to be hypomethylated in this study have not been previously investigated in SSc and SLE, though are known to be hypermethylated in cancer [16,53,54]. Finally, there are significant methylation differences in individual genes between the two patient groups, and though the methylation status of some may not conform to the majority of literature reports this does not defy what is already known about the role of these genes in SLE and SSc, which is their involvement in inflammation, autoimmunity and/or fibrosis. Moreover, Wiley *et al*., [55] suggest that variation in epigenetic changes may play a critical role in the different manifestations of the disease observed among ethnic groups. Kozłowska *et al*., [56] also suggest that exposure to unique environmental factors as well as genetic variation associated with the special racial properties of the examined groups both contributing to SLE and SSc in this population could have resulted in these deviations. It would be interesting to investigate the influence of ethnicity and/or race on the epigenetics of these genes to complete our study [57]. Hughes and Sawalha [58] suggest that the fact that patients experience periods of calm punctuated by disease flare-ups in SLE and SSc is as a result of epigenetic states which vary with time and between cell types, and if this is the case these results could be highly influenced by the disease activity state at the time of sampling. It should also be noted that since blood samples were collected only once, at commencement of the study, it was not possible to determine the effect of immunosuppressive treatment on methylation in the study subjects. As a result, neither global DNA hypomethylation nor changes in gene-specific DNA methylation patterns were accounted for by the type of medication the patients were taking. However, certain studies have reported that the antiproliferative drugs commonly used to treat SLE do not have any effect on DNA methylation [59-61], and therefore it is believed that changes in the methylation status of these patients are unlikely to be the result of immunosuppressive treatment. On the other hand, other studies show that systemic steroid use is associated with variable DNA methylation patterns throughout the genome [23,62], which could have been missed due to the limited number of genes investigated in this study.

Due to the small number of genes analysed it was not possible to neither reliably distinguish SSc from SLE, nor clearly indicate the overlap genes. It should also be noted that inconsistencies in literature on methylation/disease relationship are often attributable to the measurement method; hence the need for standardization of methods for DNA methylation analysis if further studies are undertaken. Therefore, one could say that the current data strongly demonstrate the need for investigation on a larger scale, with many subjects and many genes as is possible with the use of microarrays in genome-wide association studies. May be these epigenetic changes can be used as biomarkers of these diseases or their severity. It is also worth noting that the overall picture regarding epigenetic control of autoimmunity still remains elusive. However, evidence points to metabolic control as the central mechanism underlying aberrant gene expression leading to dysregulation of the immune system, especially in SLE [63]. Apparently mitochondrial dysfunction in T cells as a result of oxidative stress is the driver of chronic inflammation which in turn triggers autoimmunity in SLE. It is therefore possible that the metabolic profile could actually be the determining factor with regard to the differences between the different autoimmune diseases and their variations, as well as between the healthy and the sick.

## Conclusion

Notwithstanding the limitations of the study, this work confirms that SSc and SLE patients have a higher global hypomethylation than healthy subjects. As far as the authors are aware, this is the first report of a comparison of DNA methylation of genes affected in both SLE and SSc. Even though it is generally accepted that in addition to genetic dysregulation epigenetic modification of genes is responsible for both diseases the origin of epigenetic similarity of their symptoms has never been investigated. Moreover, this is the first study in which DNA methylation analysis of SLE and SSc has been done in the black African population. The study has shown that in addition to the commonly known hypomethylated genes in SLE and SSc, there are other hypomethylated genes that have not previously been investigated in both conditions, even though they are known to be hypermethylated in cancer. These genes are involved in either collagen synthesis, inflammatory response or have tumour suppressor activities, and their dysregulation could contribute to inflammation/fibrosis/tumourigenesis processes characteristic of SLE and SSc. It is obvious that there are several emergent phenotypes in SLE and SSc, likely as a result of their interaction over time which may in turn be influenced by co-occurrence with other diseases, unique environmental exposures or ethnicity/race, and it is not as yet possible to understand clearly delineation of these various components. Perhaps, it is more studies like this that could lead to identification of critical genes common to both SLE and SSc, which could possibly enable researchers to elucidate the aetiology of both diseases and to design appropriate gene targeted therapy.

## Methods

### Patients

This study was approved by the Ethics Committee for Research on Human Subjects of both the University of the Witwatersrand (Johannesburg, South Africa) and the South African National Blood Transfusion Services (SANBS). A total of 100 adult Black South African participants (18 years or older) comprising 30 patients each of SLE and SSc, and 40 healthy controls were recruited for the study. These SSc and SLE patients each fulfilling the American College of Rheumatology (ACR) criteria for SSc or SLE [64, 65] were recruited from the Connective Tissue Diseases Clinic, Chris Hani-Baragwanath Hospital (Johannesburg, South Africa). The healthy individuals were volunteer blood donors recruited during a blood drive by the SANBS (Johannesburg, South Africa).

### Serology tests and disease activity

All patients underwent baseline investigations for haematological and biochemical parameters, chest radiograph and electrocardiogram. Autoantibodies (e.g. ANA, anti-dsDNA, ATA) were detected by indirect immunofluorescence test. Various manifestations were categorized after detailed clinical examination and laboratory investigations (Table 3). Disease activity was assessed by the physician global assessment (PGA) score as well as the SLE Disease Activity Index (SLEDAI).

### Sample collection and DNA preparation

After obtaining written informed consent from all blood donors, 5 ml samples of peripheral blood were collected in EDTA tubes. Genomic DNA (gDNA) from whole blood was isolated using GenElute mammalian DNA extraction kit (Sigma-Aldrich, catalogue no. G1N70). The isolated DNA was quantified using the Nanodrop 2000C spectrophotometer (Thermoscientific) and thereafter stored at −20 °C until use.

### Quantification of global DNA methylation

Imprint™ Methylated DNA kit (Sigma-Aldrich, catalogue No. MDQ1) was used to determine global DNA methylation shifts and triplicate values were measured. This is an ELISA-based procedure consisting of four steps. Up to 200 ng of purified DNA is bound to the wells of the assay strip. The methylated DNA is detected using the capture and detection antibodies, and then quantified colorimetrically. The amount of methylated DNA present in the sample is proportional to the absorbance measured and is expressed as a percentage of the provided DNA control.

### Gene-specific DNA methylation analysis

The panel of genes profiled consisted of twelve genes, namely; *ITGAL, PRF1, SOCS1, CTGF, CXCR4, THY1, MT1G, FLI1, P16*^*INK4*^*, DNMT1, FOXP3, and CD70*. PCR Array analysis was performed using the EpiTect Methyl PCR Arrays technology (SABiosciences) on a 7300 Applied Biosystems real-time PCR instrument.

### Data analysis

The methylation qPCR Arrays data was analyzed using an integrated Excel-based template provided by the Methyl Screen™ technology (SABiosciences). The template automatically performs all ΔCt based calculations from the raw threshold cycle (Ct) values to determine gene specific DNA methylation status, and then normalizes the Ct values of both digests with the mock digestion values to calculate and report the percentage of the DNA that is methylated and unmethylated.

### Statistical analysis

The methylation results data was captured in standard data entry software STATA®. Analysis was carried out using both Kruskal Wallis test and Mann–Whitney *U*-test. Analysis of variance was conducted and Bartlett’s test was used to confirm observed variances. A Bonferroni correction was used to compare the individual groups. P values were generated from individual t-tests and differences were considered significant at a p value <0.05.

## Abbreviations

DNA: Deoxyribonucleic acid
ELISA: Enzyme-linked immunosorbent assay
EDTA: Ethylenediaminetetraacetic acid
gDNA: Genomic DNA
PCR: Polymerase chain reaction
SANBS: South African National Blood Transfusion Services
SLE: Systemic lupus erythematosus
SSc: Systemic sclerosis
ACA: Anticentromere autoantibody
ANA: Anti-nuclear antibodies
ATA: Antitopoisomerase antibodies
DLE: Discoid lupus erythematosus
ILD: Interstitial lung disease
dcSSC: Diffuse cutaneous systemic sclerosis
lcSSc: Limited cutaneous systemic sclerosis
UCTD: Undifferentiated connective tissue disease
SLEDAI: Systemic Lupus Erythematosus Disease Activity Index
PGA: Physician Global Assessment
